# Reliable Template Matching for Image Detection in Vision Sensor Systems

**DOI:** 10.3390/s21248176

**Published:** 2021-12-07

**Authors:** Youngmo Han

**Affiliations:** Department of Computer Engineering, Hanyang Cyber University, 220 Wangsimni-ro, Seongdong-gu, Seoul 04763, Korea; ymhan123@hanmail.net

**Keywords:** image processing, template matching, vision sensor

## Abstract

Template matching is a simple image detection algorithm that can easily detect different types of objects just by changing the template without tedious training procedures. Despite these advantages, template matching is not currently widely used. This is because traditional template matching is not very reliable for images that differ from the template. The reliability of template matching can be improved by using additional information (depths for the template) available from the vision sensor system. Methods of obtaining the depth of a template using stereo vision or a few (two or more) template images or a short template video via mono vision are well known in the vision literature and have been commercialized. In this strategy, this paper proposes a template matching vision sensor system that can easily detect various types of objects without prior training. To this end, by using the additional information provided by the vision sensor system, we study a method to increase the reliability of template matching, even when there is a difference in the 3D direction and size between the template and the image. Template images obtained through the vision sensor provide a depth template. Using this depth template, it is possible to predict the change of the image according to the difference in the 3D direction and the size of the object. Using the predicted changes in these images, the template is calibrated close to the given image, and then template matching is performed. For ease of use, the algorithm is proposed as a closed form solution that avoids tedious recursion or training processes. For wider application and more accurate results, the proposed method considers the 3D direction and size difference in the perspective projection model and the general 3D rotation model.

## 1. Introduction

Template matching is a well-known technique often used in many image-processing and computer vision tasks, e.g., object detection and recognition [1,2]. This is due to the simplicity and efficiency of the method. In addition, compared with learning-based methods e.g., deep-learning, NCC, and Viola-Jones. Object detection using template matching is often preferred in applications where the detection is required to perform using a single template supplied by the user and off-line learning every possible object class is impossible. Moreover, the template is unknown by the detection system beforehand [2]. The template matching method can be easily applied to various objects by changing only one template.

Template matching is simple, and suitable for VLSI logic gate implementations that achieve lower prices and faster speeds than software implementations on PC or embedded systems [3].

Considering the advantages of template matching, this paper aims to develop a template matching system for multi-purpose image detection and recognition that can be easily applied to various objects.

Despite the advantages, template matching needs some improvement, and research has been conducted to this end. Typical distortion measures for template matching can be combined with tone mapping to handle illumination-changes [4], with asymmetric correlation to handle noise [5], or with edge orientation in complement with spatial information to handle cluttered images [6].

Many studies on fast template matching methods have been reported, which focus on improving the search algorithm [7], reducing the number of matching points, or based on two state coarse fine approaches [8]. However, all the methods mentioned so far assume only 2D translation between the template and the target image. Since 2D translation alone cannot cope with local deformations of the shape of an object, pixel-by-pixel differences occur at the corresponding points of the template and query regions.

To cope with this local deformation, much research has been done. Bai et al. used “shape band”, a dilated version of templates [9]. However, the shape band does not constrain the location of the template points on the same shape. In [10], color histogram matching is used (for detection). This does not restrict the geometric transformation; however, in many cases the color histogram is not a good representation, e.g., in the presence of background clutter and occlusions [11].

Korman et al. [12] introduced a template matching algorithm under 2D affine transformation. Tian and Narsimhan [13] found a globally optimal estimation of nonrigid image distortions. In [14], viewpoint-invariant (rotation-invariant) template matching methods were intensively discussed. These methods typically use 2D affine transformations to simulate the view of the query image from different perspectives. In [1], the previous image information PA was used to improve template matching performance. PA (previous area of the object) was 2D-resized and related to NA (new area of the object). However, these methods assumed a one-to-one mapping between the template and the query region for the underlying 2D transformation.

Since the real object is 3D transformed, its imaged transform is different from the pure 2D transformation model. Due to this, an error occurs between the value predicted by the 2D transformation model and the actual value of the search image. This error decreases the accuracy of template matching.

As a solution to this problem, this paper proposes to replace the 2D transformation model of the existing method with a perspective projection of the 3D transformation model as it is. The projected 3D transformation model can further reduce template matching errors, and reduce sensitivity to size differences and 3D direction differences caused by 3D deformation of the target.

The proposed method only requires one depth image template to extend the 2D transformation model of the existing method to the projected 3D transformation model. The process of acquiring the depth image template was previously performed separately from the template matching, so it does not need to be performed in real time.

Currently, inexpensive 3D cameras such as the Kinect [15] have become popular in 3D scene modeling and human-computer interfaces (HCI) that use perceived depth information. Such devices make it easy and affordable to capture a depth image with its associated color image [16].

If the user does not have such a device, the depth image can be calculated from one short video or 2D image shots of the target using one of the existing 3D reconstruction algorithms. In this process, the existing 3D reconstruction algorithm can be based on image registration using feature descriptors such as SIFT, SURF, and so on. Existing 3D reconstruction algorithms are well known in the literature [17]. Thus, this paper focuses on designing a template matching process.

The proposed template matching process is designed as a closed-form algorithm rather than a numerical method for convenience of use. For wider applications and more accurate results, the proposed method is designed to employ a perspective projection model and a general 3D rotation model as opposed to the other approaches, in which the perspective projection model is replaced by an orthographic projection model and the general 3D rotation model is replaced by 2D planar motions or a confined-form 3D motions for a simpler formulation.

In addition, the distortion measure of the proposed template matching can be improved by combining with known methods. For example, it can be combined with [4] to increase the robustness to illumination changes, ref. [5] to increase the robustness to noise, ref. [6] to effectively handle cluttered images, and [1,7,8] to increase the speed. As suggested in [3], the proposed template matching method can be faster and lower in cost through VLSI logic gate implementation.

In terms of image detection, the biggest advantage of template matching is that it can detect various types of objects without tedious pre-training or descriptor redesign. Another advantage of template matching is that the algorithm is simple and regular, allowing not only microcomputer embedded system implementations, but also gate-level VLSI implementations. Gate-level VLSI implementations are known to be faster and cheaper than microcomputer embedded system implementations. Inheriting the advantages of template matching, this paper proposes a template matching vision sensor system for image detection that can be used for various types of objects without any prior training process or descriptor redesign. In addition, in order to increase the reliability of image detection when the posture of the object in the given image is different from the template of the object, this paper devises a method that adjusts the template by itself. For ease of use, the template matching process combined with the template adjustment process is designed as a closed algorithm without the tedious recursive process.

## 2. Proposed Template Matching Algorithm with Pose Mismatch Compensation

**Figure 1:**
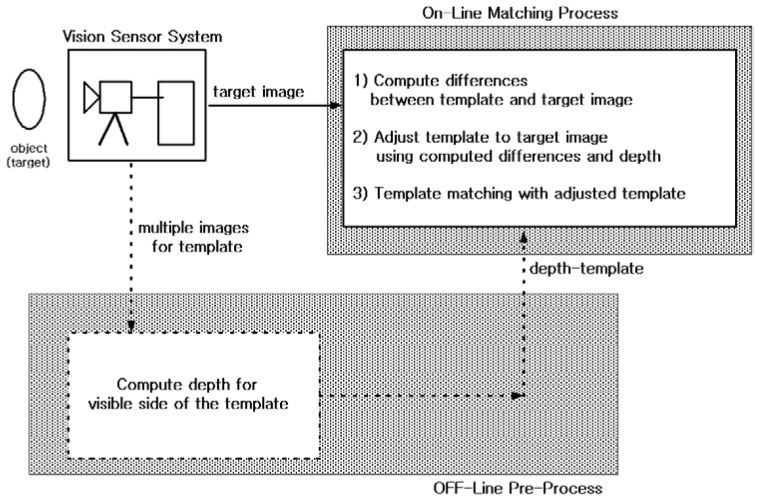
Proposed template matching vision system.

Figure 1 shows the proposed template matching vision sensor system.

The depth template is obtained as an offline process before the online matching process. The depth template can be obtained using vision sensor-based hardware (e.g., 3D depth camera, Kinect) or a reconstruction algorithm from multiple images. Multiple images can be obtained from a multi-vision sensor system, or from a sequence of images in mono vision. This off-line pre-process is well known in the vision literature. Therefore, this section focuses on designing an online matching process that is robust to pose mismatch between template and image. Pose mismatch includes both direction (viewing angle, rotation) and size (distance) differences.

Template matching is the process of finding the location of a sub image called a template within a target image (matching image, search image). This method is a simple algorithm for measuring the similarity between the template image (T) and the part of the target image (I). The basic template matching algorithm consists of calculating a distortion measure (objective function, cost function) that measures the similarity between the template and the image at each position of the target image. The minimum distortion or maximum correlation position is then taken to locate the template into the target image [18]. Representative of typical distortion measures is the sum of squared differences (SSD) described in Equation (1).

(1) $$J(d)=\sum_{q_{i}}^{}{\left(I(q_{i}+d_{})-T(q_{i})\right)}^{2}$$

where $q_{i},i=1,\cdots ,M$ are the image positions (pixel coordinates) of the template and M is the number of image positions. M and I are image intensity maps of the template and the target image, respectively. The SSD is commonly known as block matching approach [19].

**Figure 2:**
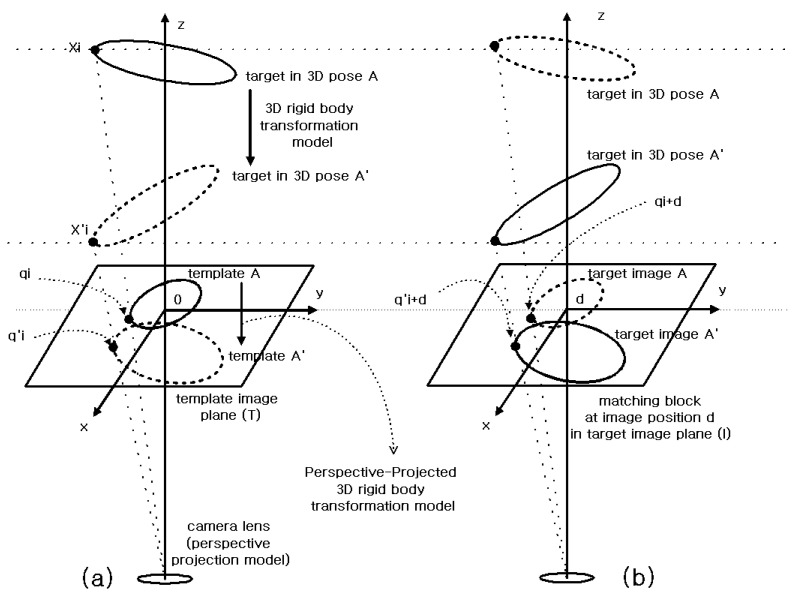
Perspective-Projected 3D rigid body transformation model for block matching of template A to target image A’ ($X\prime_{i}-X_{i}=\Delta X_{i}$, $q\prime_{i}-q_{i}=\Delta q_{i}$): (**a**) template A is a photograph of a target with a 3D pose A. (**b**) target image A’ is a photograph of target with a 3D pose A’.

To further analyze the SSD measure given in Equation (1), let us look at Figure 2. Consider a case where template A is block matched to target image A. This case shows an example where the pose of the target on the template image plane (T) matches the pose of the target on the target image plane (I). In this case, the corresponding point of $q_{i}$ on the template image plane is $q_{i}+d$ on the target image plane. That means $T(q_{i})=I(q_{i}+d)$. In this sense, SSD distortion measures are defined in the form given in Equation (1).

Consider a case where template A is block matched to target image A’. This case shows an example where the pose of the target on the template image plane (T) does not match the pose of the target on the target image plane (I). In this case, the corresponding point of $q_{i}$ on the template image plane is $q\prime_{i}+d$ on the target image plane, not $q_{i}+d$. That means $T(q_{i})=I(q\prime_{i}+d)$. In this sense, SSD distortion measures in Equation (1) can be rewritten into Equation (2):

(2) $$J(d)=\sum_{i=1}^{M}{\left(I(q\prime_{i}+d)-T(q_{i})\right)}^{2}\;\mathrm{where}\;q\prime_{i}=\psi (q_{i},\eta )$$

Here $q\prime_{i}$ can be parameterized using a transformation model, i.e., $q\prime_{i}(\eta )=\psi (q_{i},\eta )$ where η is the parameter vector of the transformation model ψ. Finally, the value of d that minimizes J(d) is the matching position.

From the point of view of Equation (2), the conventional SSD given in Equation (1) can be regarded as an approximation of Equation (2), assuming the transformation model $\psi (q_{i},\eta )\approx q_{i}$. This transformation model assumes that the template and the target image have the same pose. As a result, this transformation model of the conventional SSD does not fit well when the poses of the template and target image are different.

This weakness can be compensated for by using a more sophisticated transformation model. For this purpose, well-known 2D transformation models, such as 2D homogeneous transformation, affine transformation, and similar, can be used. However, if the target is an object undergoing 3D transformation, there is a limit to applying a simple 2D transformation model. As shown in Figure 2, the target performs a 3D transformation. The projection of these 3D transformations onto the image plane does not fit well with a simple 2D transformation model.

For example, 3D rotations and translations perpendicular to the image plane cause deformations of size and orientation that cannot be correctly described using simple 2D translations or 2D transformation models. To properly describe these deformations, it is necessary to take into account the original 3D motion.

With this in mind, this paper does not forcibly approximate a 3D transformation of a target to a simple 2D transformation, but expresses it as a 3D rigid body transformation model as it is. The 3D rigid body transformation model is then projected onto the image plane using a perspective projection camera model. The perspective projected 3D rigid body transformation model thus obtained is adopted as a transformation model between the template and the target image. Thus, the proposed matching measure (objective function) is an SSD whose transformation model, i.e., $q\prime_{i}(\eta )=\psi (q_{i},\eta )$ has been upgraded with perspective projected 3D rigid body transformation (perspective projection of a 3D rigid body transformation model).

From now on, we will formulate the proposed perspective-projected 3D rigid body transformation model of the target.

### 2.1. 3D Transform Model of Target

We begin by formulating a 3D rigid body transformation model of the target. In Figure 2, suppose the difference between the two poses of a given target, 3D pose A (pose of the target in the template) and 3D pose A’ (pose of the target in the target image), consists of rotation by an angle θ∈R about the axis of rotation $w\in R^{3}$ and translation by $P\in R^{3}$. If the coordinate of a point on the target is $X_{i}\in R^{3}$ when 3D pose is A and is $X\prime_{i}\in R^{3}$ when 3D pose is A’, the relationship as shown in Equation (3) holds.

(3) $$X\prime_{i}=RX_{i}+P$$

Here $R=\exp([\hat{w}]\theta )$ is the rotation matrix and $\hat{w}={\left[\begin{array}{ccc} {\hat{w}}_{1} & {\hat{w}}_{2} & {\hat{w}}_{3} \end{array}\right]}^{T}$ is the unit vector in the direction of w. The notation $\left[\hat{w}\right]$ is defined as the following skew-symmetric form:

(4) $$[\hat{w}]=\left[\begin{array}{ccc} 0 & -{\hat{w}}_{3} & {\hat{w}}_{2}\\ {\hat{w}}_{3} & 0 & -{\hat{w}}_{1}\\ -{\hat{w}}_{2} & {\hat{w}}_{1} & 0 \end{array}\right]$$

If $\left|\theta |<<1[rad]\approx 57.3[\deg\right]$, then we can approximate:

(5) $$\exp([\Phi ])\approx I+[\Phi ]\;\mathrm{where}\;\Phi =\hat{w}\theta$$

Using Equation (5), we obtain:

(6) $$\Delta X_{i}=X_{i}^{\prime }-X_{i}=[\Phi ]X_{i}+P$$

### 2.2. Projection of 3D Transfromation

We will now consider the projection of the 3D rigid body transformation given in Equation (6) onto the image plane. Suppose the template and the target images are photographs obtained by shooting the target with a perspective projection camera. In this process, 3D coordinates $X_{i}\in R^{3}$ and $X\prime_{i}\in R^{3}$ are projected to image coordinates $q_{i}$ and $q\prime_{i}{}^{3}$, respectively.

By using the perspective projection model, we can relate $X_{i}$ and its corresponding image coordinate $q_{i}$ as follows:

(7) $$q_{i}={\left[\begin{array}{ccc} q_{ix} & q_{iy} & 1 \end{array}\right]}^{T}=s_{i}X_{i}$$

Here, *T* denotes transpose, $s_{i}=1/z_{i}$ and $z_{i}$ is the scaled depth (the third component of point $X_{i}$ divided by the focal length f of the camera).

Because $q_{i}^{}=s_{i}X_{i}$, we calculate:

(8) $$\Delta q_{i}^{}=\Delta s_{i}X_{i}+s_{i}\Delta X_{i}$$

Because $s_{i}=1/z_{i}$, we calculate:

(9) $$\Delta s_{i}^{}=-\Delta z_{i}/z_{i}^{2}=-s_{i}^{2}\Delta z_{i}=-s_{i}^{2}(e_{3}^{T}\Delta X_{i})$$

By substituting Equation (9) into Equation (8) and using Equation (7), we obtain:

(10) $$\Delta q_{i}^{}=s_{i}(I-q_{i}e_{3}^{T})\Delta X_{i}$$

Substituting Equation (6) into Equation (10), and using Equation (7) and the relation: $[\Phi ]q_{i}=-[q_{i}]\Phi$, we obtain:

(11) $$\Delta q_{i}^{}\equiv q\prime_{i}-q_{i}=F_{i}\eta$$

where $F_{i}=\left[\begin{array}{cc} -(I-q_{i}e_{3}^{T})[q_{i}] & s_{i}(I-q_{i}e_{3}^{T}) \end{array}\right]$, $\eta =\left[\begin{array}{c} \Phi \\ P \end{array}\right]$

### 2.3. Proposed Matching Measure

If the displacement of the image coordinates is not large, i.e., ($\left||\Delta q_{i}^{}||<<||q_{i}|\right|$), the integrand of the SSD matching measure given in Equation (2) can be approximated by Equation (12):

(12) $$I(q_{i}+\Delta q_{i}^{}+d)\approx I(q_{i}+d)+{\left(\partial I/\partial q_{i}^{}\right)}_{q_{i}+d}^{T}\Delta q_{i}^{}$$

Here, we define $\partial I/\partial q_{i}^{}$ as a column vector and *T* denotes the transpose.

Substituting Equations (11) and (12) into the SSD matching measure given in Equation (2), we get the proposed matching measure as Equation (13):

(13) $$J(\eta )=\sum_{i=1}^{M}{\left(a_{i}+B_{i}\eta \right)}^{2}$$

where $a_{i}=I(q_{i}+d)-T(q_{i})$ and $B_{i}={\left(\partial I/\partial q_{i}^{}\right)}_{q_{i}+d}^{T}F_{i}$.

### 2.4. Proposed Algorithm Summary

Solving the first order necessary conditions for an optimization, i.e., ∂J/∂η=0, the value η that minimizes Equation (13) is given by Equation (14):

(14) $$\eta =-(\sum_{i=1}^{M}B_{i}{}^{T}B_{i}{)}^{-1}(\sum_{i=1}^{M}B_{i}{}^{T}a_{i})$$

By combining Equations (13) and (14), the proposed template matching procedures can be summarized as follows.

**Figure 3:**
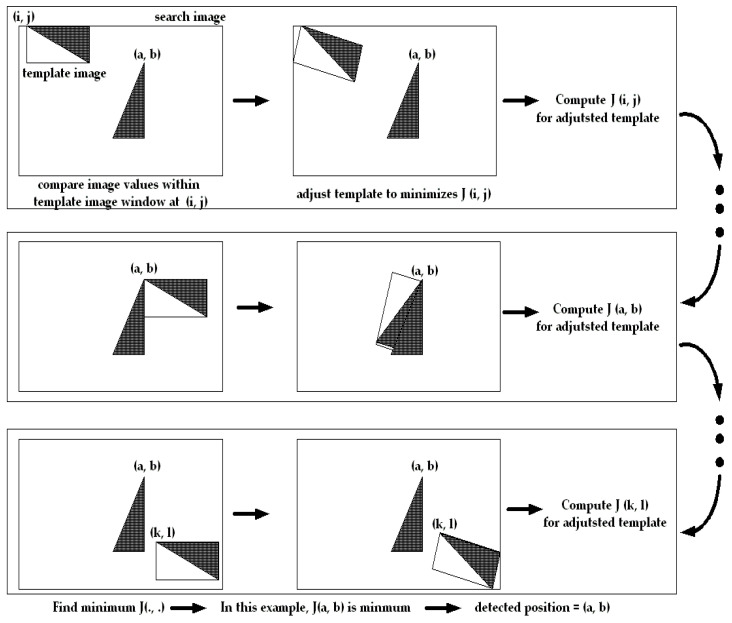
A test image of finding a right triangle with a different 90-degree angle from the template. Each step of Algorithm 1 was applied to the test image. The template window is moved to each pixel coordinate of the search image to analyze the image intensity distribution within the template window. (The first, second, and third lines describe the case of the template window at (i, j), (a, b), and (k, l), respectively.) Step 1 calculates the adjusted template parameters by analyzing the image intensity distribution of the template window at each position. As a result, we have the effect of rotating the template window at each position as shown in the middle figure of each row. Step 2 computes the matching measure value J for the rotated template at each position. Step 3 selects the minimum value among the matching measure values calculated at each position. In this test image, J (a, b) is minimal. Then the matching position will be (a, b).

For better understanding, a diagram showing the results of each step by applying Algorithm 1 to the test image is given below. Figure 3 is a test image to find a right triangle with a different angle of 90 degrees from the search image using the template for the right triangle. Looking at Step 1 of Algorithm 1, the template image window is moved to each pixel coordinate of the search image, and the image intensity distribution within the template image window at the current position is analysed. The figure on the left in the first row of Figure 3 shows the case where the template window is moved to the pixel coordinate (i, j). Visualizing this, as shown in the middle figure, the template’s posture will be expressed in the adjusted form at pixel coordinate (i, j). Step 2 of Algorithm 1 uses the adjusted template parameters to calculate the matching measure value J (i, j) at pixel coordinate (i, j). Visualizing this, the matching measure value J (i, j) at pixel coordinate (i, j) is calculated using the image intensity distribution within the adjusted template window, not the original template window.
**Algorithm 1.** Proposed template matching procedures• For each row on the search image  For each column on the search image  - **Step 1:** Compute adjusted template parameters    $a_{i}=I(q_{i}+d)-T(q_{i})$, $B_{i}={\left(\partial I/\partial q_{i}^{}\right)}_{q_{i}+d}^{T}F_{i}$,    $F_{i}=\left[\begin{array}{cc} -(I-q_{i}e_{3}^{T})[q_{i}] & s_{i}(I-q_{i}e_{3}^{T}) \end{array}\right]$    $\eta =(\sum_{i=1}^{M}B_{i}{}^{T}B_{i}{)}^{-1}(\sum_{i=1}^{M}B_{i}{}^{T}a_{i})$  - **Step 2:** Compute the matching measure using adjusted template parameters    $J(d)=\sum_{i=1}^{M}{\left(a_{i}+B_{i}\eta \right)}^{2}$  END FOR END FOR  **Step 3:**  IF $J(row*,column*)$ is minimum THEN    $\left(row*,column*\right)$ is the matching position.    END IF

Then, move the template image window to the next pixel coordinate, and do the same. The figures in the second row show examples of doing the same thing for pixel coordinate (a, b), and the figures in the third row for pixel coordinate (k, l). Here, at each pixel coordinate, the angle at which the template is adjusted is different. This is because the image intensity distribution within the template window at its pixel coordinates is different. After calculating the matching measure value for each pixel coordinate of the search image in this way, as expressed in Step 3 of Algorithm 1, the smallest value is selected among them. For this test image, J (a, b) will be minimal. Then the matching position will be (a, b).

## 3. Results

This section verifies the performance of the proposed template matching vision system given in Figure 1. Methods of obtaining the depth of a template using stereo vision or a few (two or more) template images or a short template video via mono vision are well known in the vision literature and have been commercialized. Therefore, in this paper, we simply adopt one of these methods for the ‘OFF-Line Pre-Process’ block in Figure 1 (in this section, we apply the algorithm of [17] to a short template video to obtain depth). An experiment is used to verify whether Algorithm 1 can effectively detect the position of various posed objects in a given image. This section numerically verifies the performance of the proposed method (Algorithm 1) against orientation and size differences between the template image and the search image. These tasks are performed using a commercial numerical simulation program on a computer with a clock speed of 3 GHz and 504 MB RAM. The experiment video sequences are recorded using a monocular camera with known intrinsic parameters that are assumed to remain unchanged throughout the sequence.

### 3.1. Conceptual Comparison of Proposed Method with Contemporary Methods

**Figure 4:**
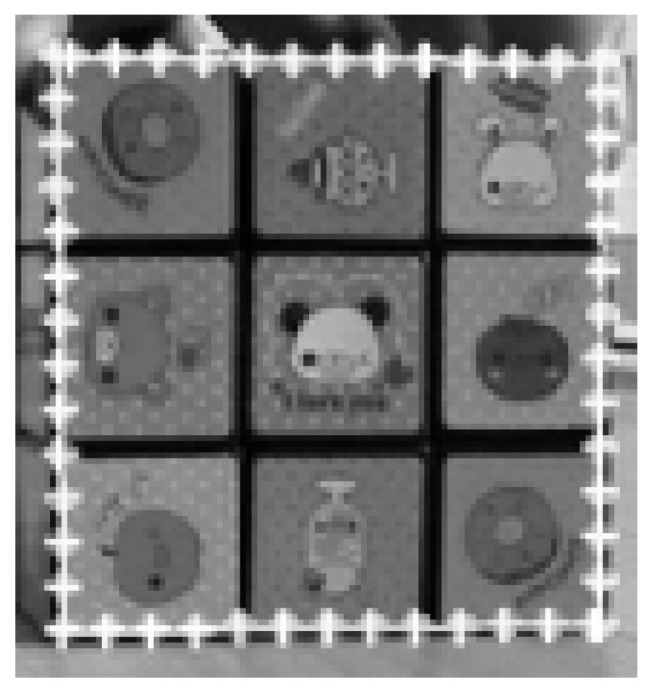

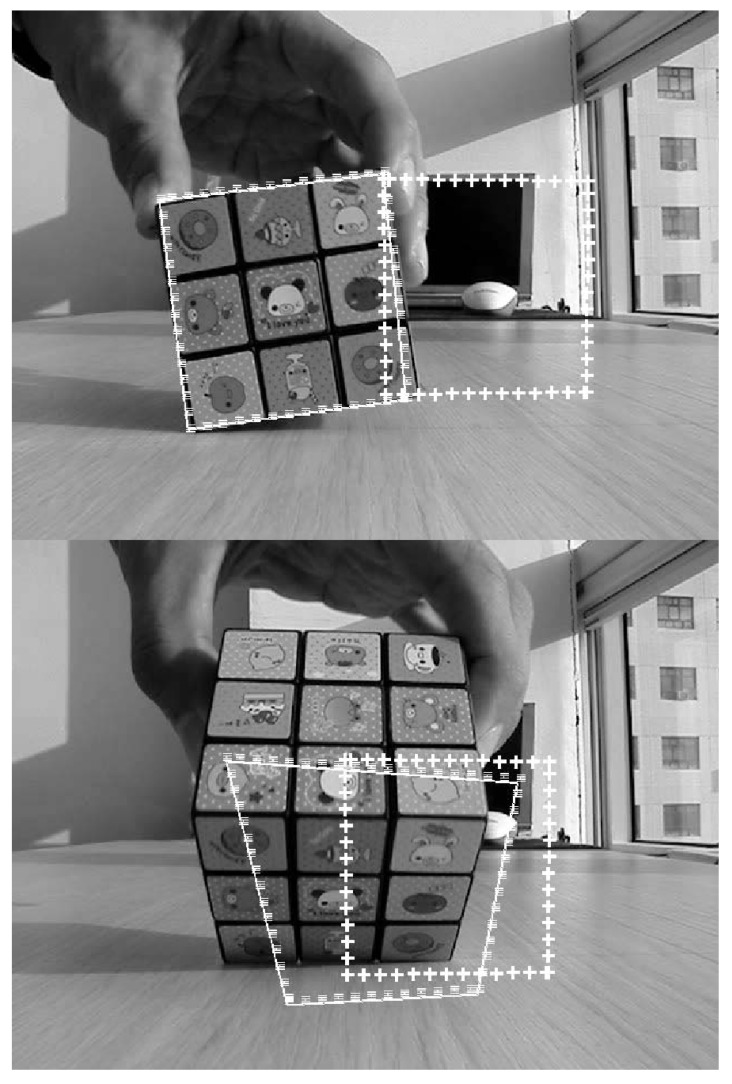

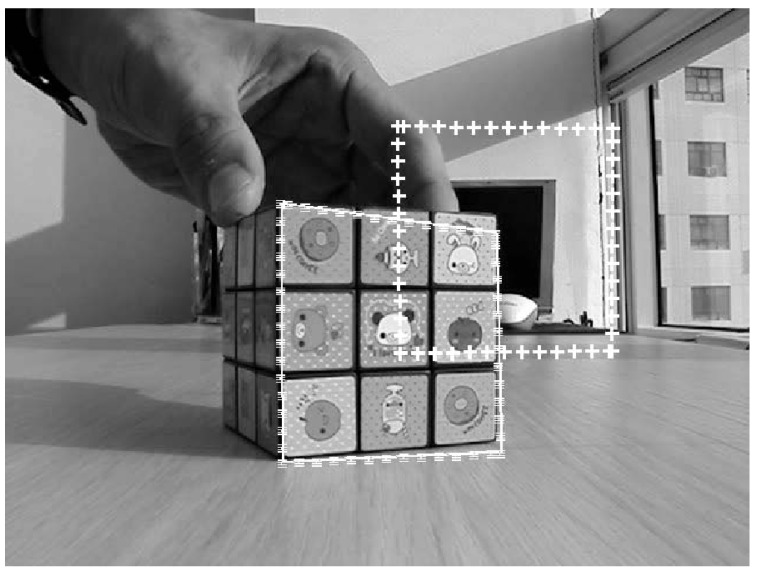
Conceptual comparison of the proposed method with contemporary methods. The leftmost picture is the given template. Areas to be used as indicators of comparison are marked with white highlight lines. In the target images given on the right, the area marked by the solid line (the proposed method) was deformed to match the target image, like the area marked by the triple line (brute-force method). On the other hand, the area indicated by the cross line (correlation method) was not deformed.

**Figure 5:**
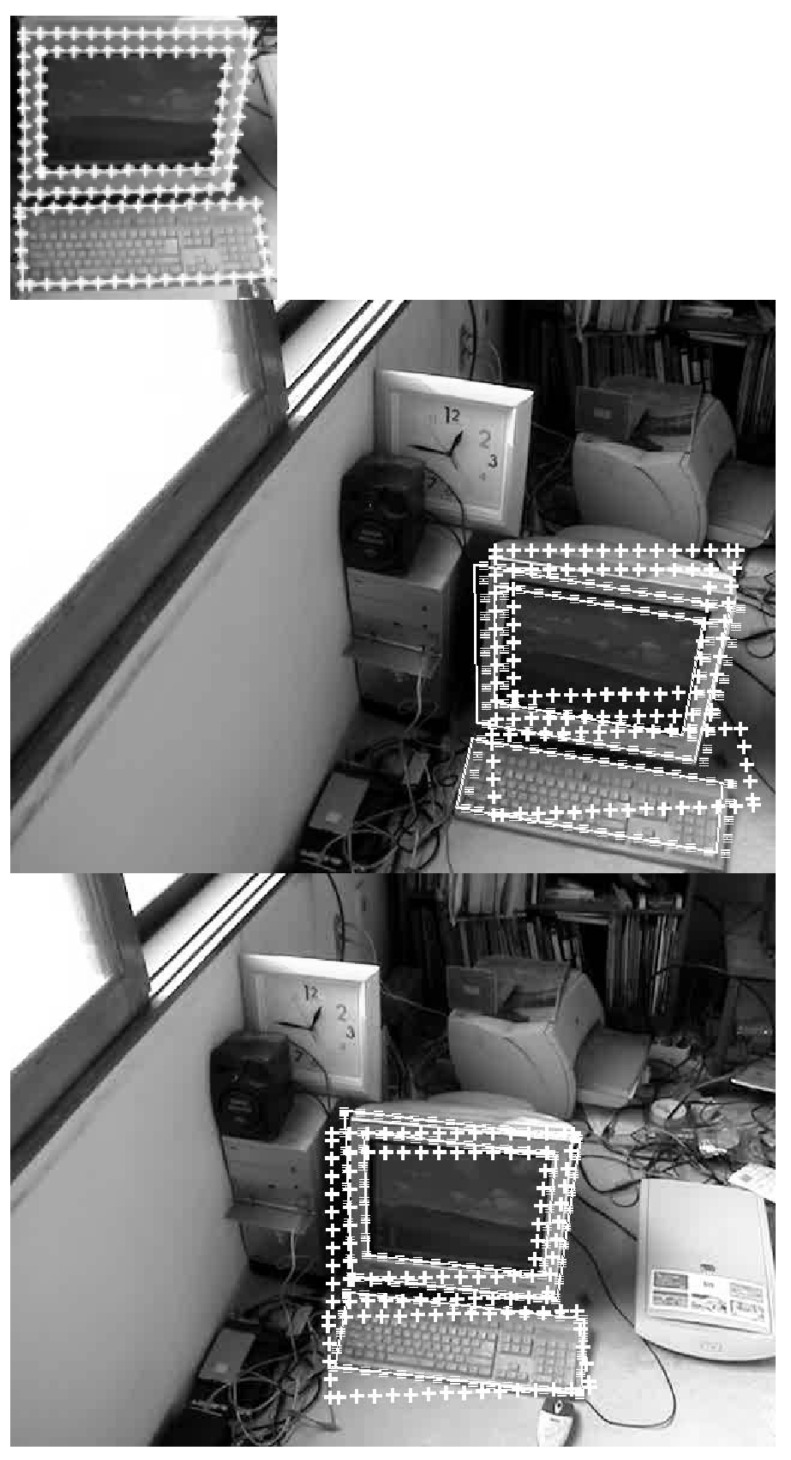

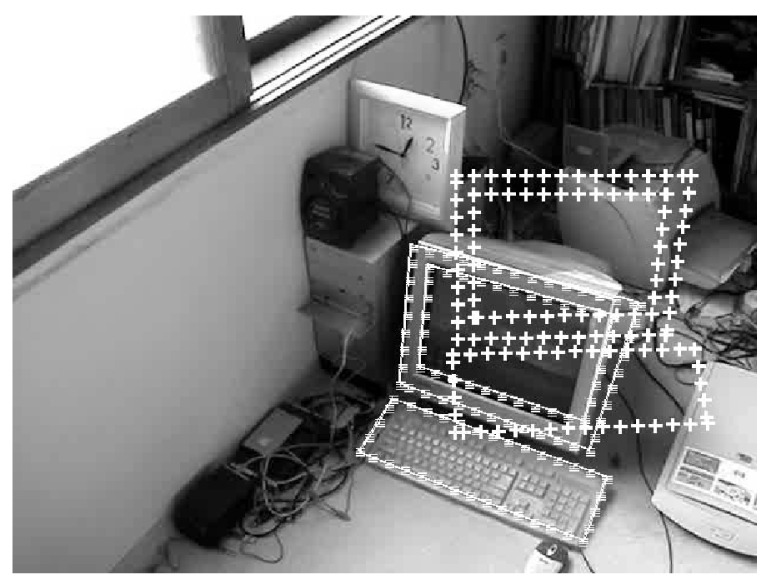
Conceptual comparison of the proposed method with contemporary methods. The leftmost picture is the given template. Areas to be used as indicators of comparison are marked with white highlight lines. In the target images given on the right, the area marked by the solid line (the proposed method) was deformed to match the target image, like the area marked by the triple line (brute-force method). On the other hand, the area indicated by the cross line (correlation method) was not deformed.

**Figure 6:**
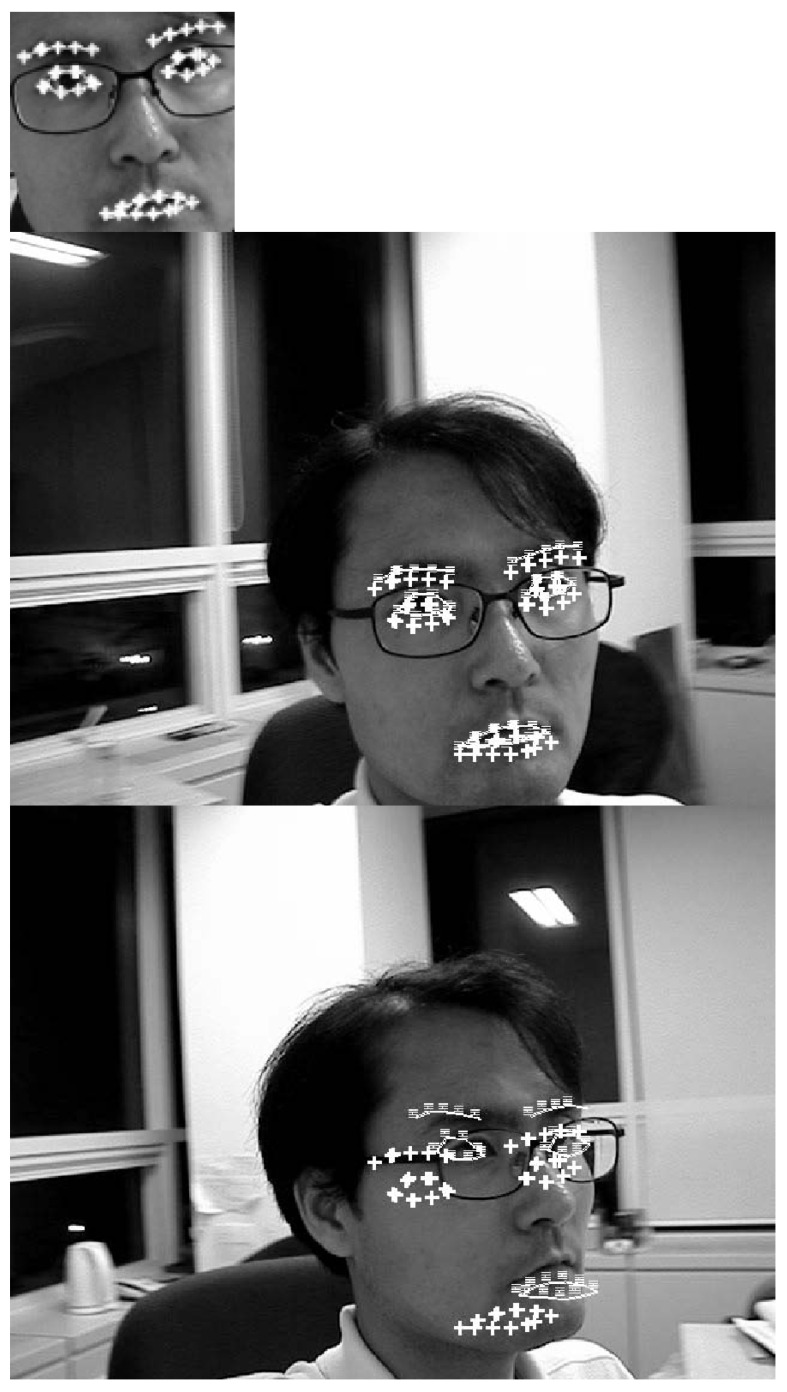

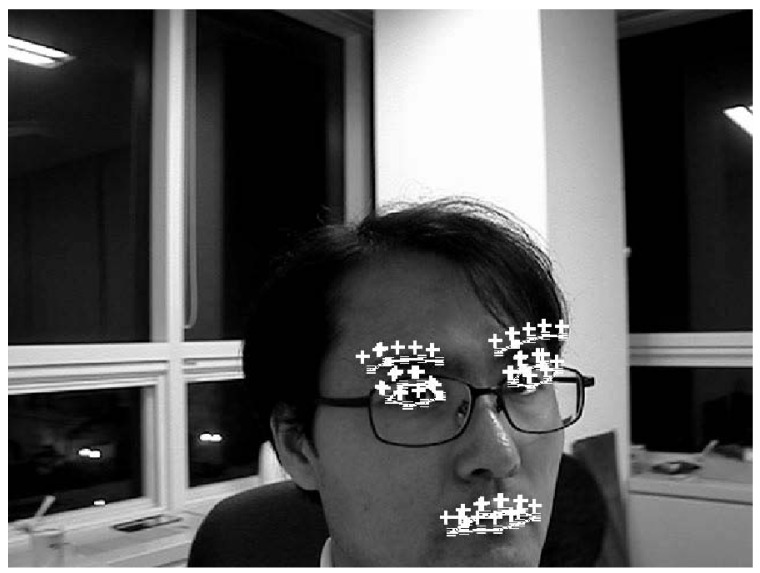
Conceptual comparison of the proposed method with contemporary methods. The leftmost picture is the given template. Areas to be used as indicators of comparison are marked with white highlight lines. In the target images given on the right, the area marked by the solid line (the proposed method) was deformed to match the target image, like the area marked by the triple line (brute-force method). On the other hand, the area indicated by the cross line (correlation method) was not deformed.

This subsection provides conceptual comparison of proposed method with contemporary methods. Figure 4, Figure 5 and Figure 6 show how the proposed method adapts a given template to the target image. The conceptual differentiation of the proposed method was highlighted by comparing it with the correlation coefficient method [20] (dotted line) and the brute-force method [21] (triple line), which are representative methods for correcting the difference in direction and size between the object template and the object in the given image. The first image in Figure 4, Figure 5 and Figure 6 is the template. To clearly illustrate the shape of the adjusted template, areas to be used as indicators of comparison are marked with white highlight lines. In the target images, the area marked by the solid line (the proposed method) was deformed to match the target image, like the area marked by the triple line (brute-force method). On the other hand, the area indicated by the cross line (correlation method) was not deformed.

In Figure 4, the target is a cube. The image size is 640 × 480. The cube in the first image is the template. The visual detection problem is to find the cube in the second, third and fourth images. The difficulty of this example is that it involves complex and abrupt 3D motions.

In Figure 4, the correlation coefficient method does not consider the pose of the subject (i.e., the pose of the template does not change according to the search image) and does not match well (i.e., matched positions of the template and the search image are remote). Even the correlation coefficient method fails to match in the second, third, and fourth frames. Contrarily, the proposed method and the brute force method consider the pose of the subject (i.e., the modified template has a pose similar to the search image) and match well (i.e., the matched positions of the template and the search image are adjacent).

Table 1 shows the matching errors of the algorithms. Table 1 shows that the proposed method reduces many the matching errors of the correlation method, and the brute force method produces just slightly fewer matching errors than the proposed method.

In Figure 5, the target is a PC. The image size is 640 × 480. The PC in the first image is the template. The visual detection problem is to find the PC in the second, third, and fourth images. In contrast to the first example scenario, in which a fixed camera records a moving object, the second example scenario is designed to treat multiple stationary objects recorded by a moving camera. While the correlation coefficient method matches well in the second and the third frames, it fails to match in the fourth frame of Figure 5.

Table 2 shows the matching errors of the algorithms. Whereas the brute force method produces the smallest matching errors, the proposed method also produces small matching errors that are close to those of the brute force method.

Using the two example scenarios, we evaluated the performance of the proposed method for various objects. The purpose of these two example scenarios is to show that the proposed method is not confined to just a human face, and is applicable to more general objects. Now, we finally consider a human face in the third example scenario, shown in Figure 6. In Figure 6, the target is a human face. The human face in the first image is the template. The visual detection problem is to find the human face in the second, third and fourth images.

Figure 6 shows the correlation coefficient method fails to match in the third frame where pose mismatches between the template and the search image are large. The proposed method and the brute force method match well in all frames.

Table 3 shows the matching errors of the algorithms. In this table, the matching errors produced by the proposed method are much less than those of the correlation method. In this table, the matching errors for the three methods are on the same order as those shown in Table 1 and Table 2.

The matching results of Figure 4, Figure 5 and Figure 6 show that the proposed method and the brute force method have almost the same accuracy. However, the proposed method has much more computational efficiency than the brute force method. The correlation method performs one-time template matching using a given template. The proposed method performs one-time template matching using the adjusted template. The brute-force method performs a large number of templates matching, transforming a given template for each possible direction and size.

### 3.2. More Systematic Comparison

For a more systematic analysis, we conduct an experiment with two open data sets (each data set contains 537 and 1344 images) and compare the results with the latest template matching method, Fast Affine Template Matching (FATM) [12]. FATM uses a 2D affine transform to improve performance for direction differences.

**Figure 7:**
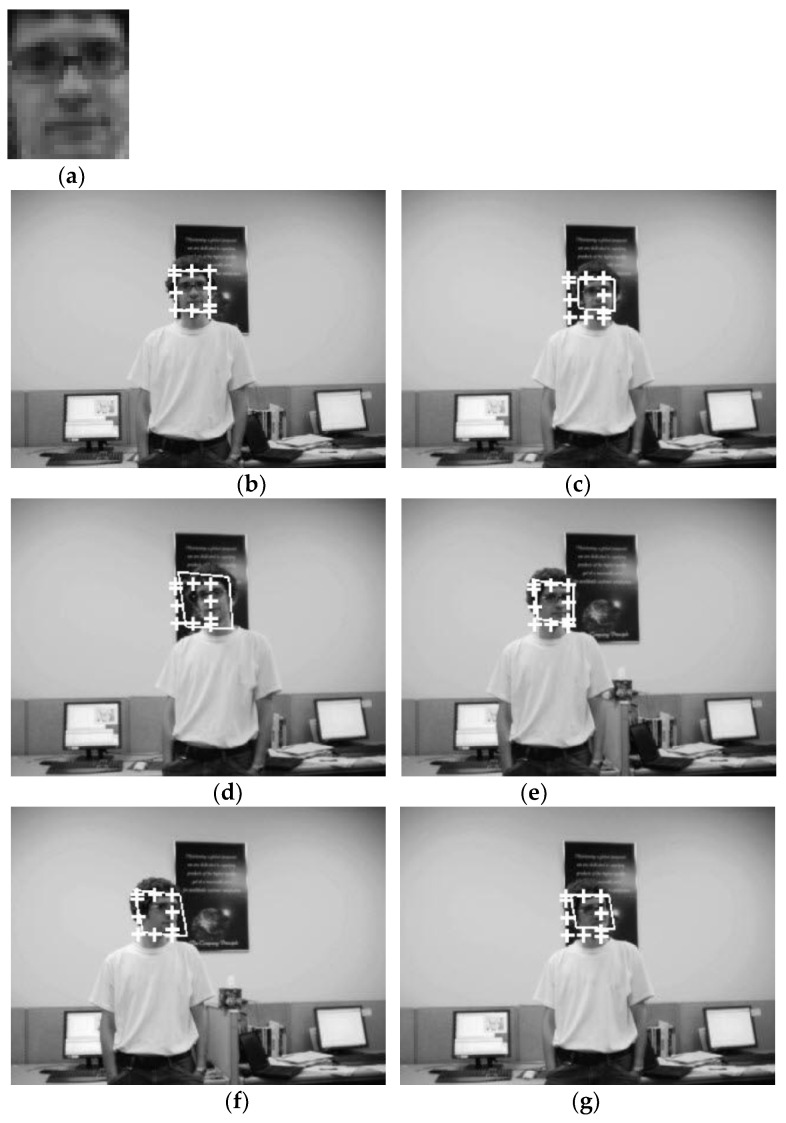
Selected results of face detection using David open data sets [22]. The first image (**a**) is the template. The face detection problem is to find the same person as the template in various images with different poses and sizes from the template, such as images (**b**–**g**).

**Figure 8:**
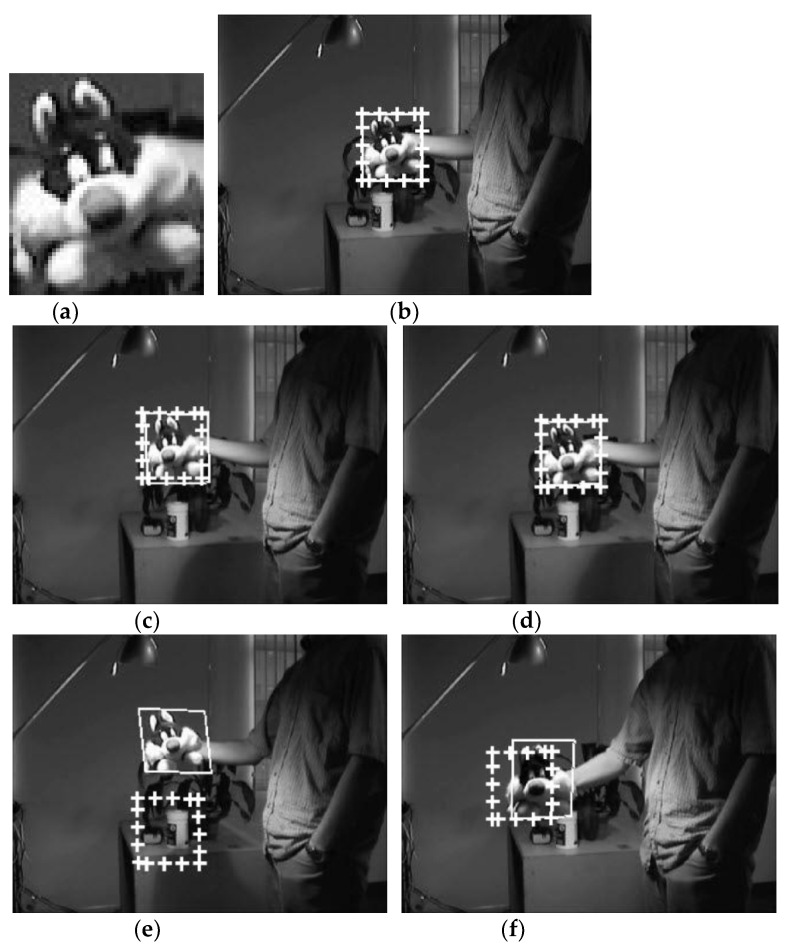

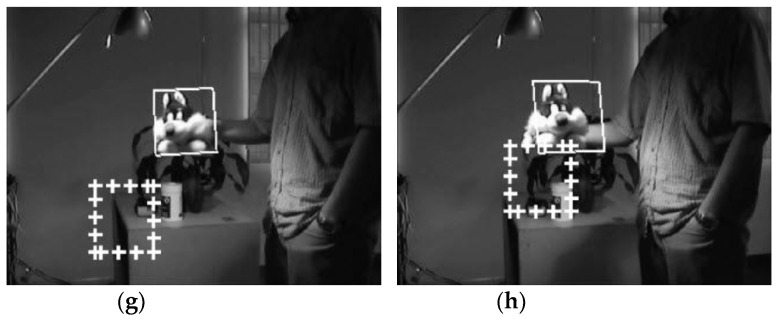
Selected results of object detection using Sylvester open data sets [23]. The first image (**a**) is the template. The object detection problem is to find the same object as the template in various images with different poses and sizes from the template, such as images (**b**–**h**).

In our experiments so far, we have used the data set we have prepared. In our experiments from now on, we will use the open standard data set. An open dataset introduces a new level of difficulty in that the experimental conditions cannot be controlled. The first open data set used in the experiment is the ‘David’ data set [22] shown in Figure 7. The second open standard data set is the ‘Sylvester’ data set [23] given in Figure 8. In the open data set, too, to emphasize that the proposed method is applicable to various types of targets, a cat doll (Sylvester) and a human face (David) were selected.

Figure 7 shows template matching for detection performed on an open data set called ‘David’ [22]. Then, this experiment searched the ‘David’ template in the target image (320 × 240 size), extracted from one of the 537 images. Figure 7a shows the template and Figure 7b–g show the selected ones of the template matching results. In Figure 7, the detected area is marked with a rectangular box as in the general detection problem. The proposed method and FATM are denoted by solid and dotted lines, respectively.

Table 4 shows the average matching errors for the entire Figure 7 experiment. Table 4 shows that the matching errors of the proposed method are much smaller than FATM.

In Figure 7b,e, the template and the matching image have similar size and pose. In this case, both methods succeeded in face detection. However, it can be seen that the positional error of the detected face obtained by FATM is larger than the proposed template matching method in the remaining matching images having a pose or size different from that of the template.

The next experiment is performed on an open dataset called ‘Sylvester’ [23] that contains 1344 pictures. Then this experiment searched the ‘Sylvester’ template in the target image (320 × 240 size), extracted from one of the 1344 images. Figure 8a shows the template and Figure 8b–h show the selected ones of the template matching results.

In Figure 8b–d, the template and the matching image have similar size and pose. In this case, both methods succeeded in object detection. However, the positional error of the detected object obtained by FATM is larger than the proposed template matching method in Figure 8c, presenting a pose different from that of the template. In Figure 8e,g,h, where the pose and size of the template and the matching image differ more, FATM fails to detect the object. In contrast, the proposed template matching method succeeded in detecting the object in all of Figure 8e,g,h.

Figure 7 and Figure 8 show that the proposed method well detected the position of the target regardless of 2D direction, 3D direction, and size differences. FATM also detected target positions well when the direction and size differences were small (see Figure 7b,e and Figure 8b–d). However, the larger the difference in direction and size, the greater the target position error than the proposed method (see the remaining figures in Figure 7 and Figure 8). This is because the underlying 2D affine transformation model of FATM has limitations in estimating the 3D transformation (combination of size, 2D and 3D direction differences) of the target, and thus an estimation error occurs. The estimation error due to the approximated model increases as the difference value increases.

Table 5 shows the average matching errors for the entire Figure 8 experiment. Table 5 shows that the matching errors of the proposed method are much smaller than FATM. The difference in matching errors is greater in the Figure 8 experiment than Figure 7 experiment. This is because the Figure 8 experiment contains more cases where object detection failed, as shown in Figure 8e,g,h.

**Figure 9:**
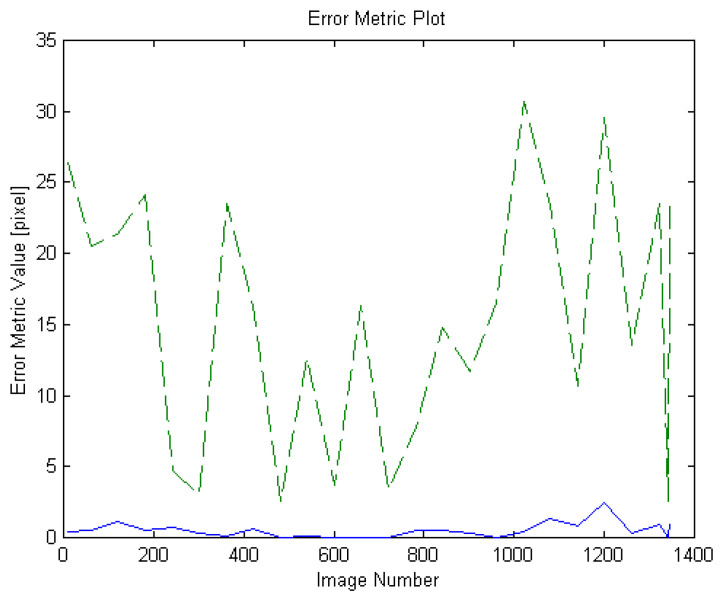
Plot of matching error metrics as a target object rotates in 3D space. (The Sylvester video given in Figure 8 was used as the selected target object for this purpose).

Figure 9 shows the result of performing template matching and plotting error metric values for images of a target object in various postures different from the template while rotating the object in 3D space. Looking at this plot, it can be seen that the proposed method has consistently fewer error values and more stable form than FATM. (The Sylvester video given in Figure 8 was used as the selected target object for this purpose).

## 4. Proposed Template Matching Method Optimized for Home Multi-Purpose Image Detection Device (Qualitative Comparison with CNN)

In the problem where the subject is limited to the face, deep learning-based methods such as CNNs are widely used for face recognition. CNN increases the recognition rate by performing pre-training on a specific object using vast image data. Therefore, it can be used only when the object is specified in advance and massive image data for the object is obtained. On the other hand, the proposed template matching method does not use a large amount of image data and can conveniently change the object at any time. Since these two methods are different types, direct comparison is difficult. Moreover, the accuracy of CNN depends on the image data used in the pre-training process of a specific object. For this reason, it is difficult to make a quantitative comparison in the theme of this study for multi-purpose image detection that changes the object at any time. However, if we qualitatively analyse the characteristics of the two methods, we can find application examples where the proposed method is better than CNN. One example is the multi-purpose image detection device for home use, which will be described in this section.

If only the face recognition rate is considered, CNN outperforms other methods. However, in order to obtain a good recognition rate, a large quantity of time, big image data, and expert’s efforts are required for pre-training the target object. Moreover, this pre-training task must be repeated every time the target changes. Such pre-training tasks are difficult for non-experts to perform at home. Due to these characteristics, when the CNN is used as home equipment, it is difficult to make it a multi-purpose device that non-experts can change the target from time to time, and it must be made in the form of an embedded system in which the target is pre-determined.

Contrarily, the template matching method can be applied to a variety of targets (individual objects, faces, etc.) without requiring any hardware or software adjustments. Therefore, there is no need for a lot of expensive hardware specialized for each type of the targets. In addition, there is no need for a specific reinstallation or reset procedure for each type of the subjects that an expert carries out.

In this respect, the proposed template matching method is more suitable for home multipurpose image detection devices than CNN, although the CNN method shows higher accuracy than the proposed method in the face recognition problem. The following points support this situation. The proposed method shows higher accuracy than the existing template matching method because the sensitivity problem for direction and size difference is compensated. Household products have a lower requirement for accuracy than military or medical applications. Instead, they are inexpensive, compact, lightweight, and convenient to use without professional care.

The multipurpose image detection device should be easily applied to various kinds of targets since the target is not limited to the face. To be applied in home appliances, the image detection device must operate in a home appliance environment, such as home PCs and home digital cameras, without specialized and expensive hardware. Given the lack of supervision of qualified workers at home, the image detection device should be easy to install and manage. When considering the low performance of home appliances, the automatic image detection system should be computationally efficient. Considering the conditions of such home-use multi-purpose image detection devices, a qualitative comparison between the proposed template matching method and CNN is as follows.

First, in order to have a good face recognition success rate, the deep learning method requires customized big data for each type of target, and pre-training using the big data is required. Although real-time speed can be achieved in the face recognition process, the pre-training process requires a lot of computation and professional management. Therefore, deep learning methods can be used only when the large data for pre-training can be obtained, and when a lot of pre-training time can be obtained. In most cases, experts are needed to oversee this complex pre-training process. These constraints can only be met mainly in laboratories or companies, but not in the average person or household.

On the other hand, the proposed template matching method only needs to change the template image when the type of the target is changed and does not require a pre-training process. Whenever the target is changed, CNN requires a huge amount of training images to model the changed target, whereas in the proposed method, two or three images are sufficient to create a template of the changed target. The usage of the proposed method is extremely simple compared to deep learning methods, and is performed automatically without the need for an expert.

Second, since the proposed method is a simple algorithm-type template matching method, it is convenient to implement with VLSI hardware. These VLSI hardware implementations offer significant speed gains, making real-time implementations easier, less expensive, and significantly smaller in size and weight.

Third, household products have a lower requirement for accuracy than military or medical applications. Instead, they are inexpensive, compact, lightweight, and convenient to use without professional care.

Currently, deep learning is also being developed in the form of embedded devices or mobile devices. However, the hardware of an embedded or mobile device consists of a microprocessor with an embedded operating system, and deep-learning algorithms are mounted in the microprocessor as application software. This form is a kind of software implementation, which is different from the hardware implementation mentioned in this paper.

In contrast, the template matching method including the proposed method may be implemented as digital logic gates of VLSI, not software mounted in a microprocessor. These VLSI digital logic gate implementations not only have significantly improved speeds thousands of times more than software implementations on microprocessors, but they are also much less expensive, extremely small and light. Thus, compared to embedded devices with deep learning methods, the VLSI digital logic gate implementation of the template matching method can greatly reduce the cost of home appliances, and it is much more advantageous to be manufactured as a wearable accessory because of its small size and light weight.

By comparing and analyzing the qualitative characteristics described above, it can be seen that the proposed template matching method is better (more suitable) for a multipurpose image detection appliance for home use.

## 5. Conclusions and Future Work

In this paper, we studied the template matching method. To reduce the sensitivity of the conventional template matching methods to pose mismatches between the template image and the matching image, the proposed method adjusts the pose of the template image to that of the matching image. By reducing errors incurred from pose mismatches between the template image and the matching image, the accuracy in the matching results is enhanced. Moreover, contrary to other contemporary methods, the proposed method does not employ any short-cut models for the projection model and the 3D rotation model. For this reason, a better matching accuracy can be obtained. Nevertheless, the additional module that adjusts the template image is designed to have a closed form. Because the additional module has a closed form, the additional increase in the computational time is simply small. Since the proposed template matching method is a region-based matching approach, it can be applied to more general objects as well as human faces, contrary to the other feature-based matching approaches.

## Figures and Tables

**Table 1:** Average position errors (in pixels) of the entire Figure 4 experiment.

Proposed	Correlation	Brute-Force
1.6832	16.8401	1.3462

**Table 2:** Average position errors (in pixels) of the entire Figure 5 experiment.

Proposed	Correlation	Brute-Force
1.9759	11.9480	1.2286

**Table 3:** Average position errors (in pixels) of the entire Figure 6 experiment.

Proposed	Correlation	Brute-Force
0.6403	2.3631	0.2332

**Table 4:** Average position errors (in pixels) of the entire Figure 7 experiment.

Experiment	Proposed	FATM
Figure 7	1.9092	6.0512

**Table 5:** Average position errors (in pixels) of the entire Figure 8 experiment.

Experiment	Proposed	FATM
Figure 8	2.2100	42.0112
